# Effects of N_2_-LED Integrated Cold Storage on Postharvest Quality Retention of Sichuan Pepper

**DOI:** 10.3390/foods15152708

**Published:** 2026-07-31

**Authors:** Kaiping Cong, Andi Suo, Tingting Li, Dandan Zhou

**Affiliations:** 1 Cixi People’s Hospital, Wenzhou Medical University, Cixi 315300, China; ckpnjfu@njfu.edu.cn; 2 State Key Laboratory for Development and Utilization of Forest Food Resources, Nanjing Forestry University, Nanjing 210037, China

**Keywords:** Sichuan pepper, LED, cold storage, polyphenol, flavonoid, flavor compounds, OH-α-sanshool

## Abstract

This study examines the effects of nitrogen (N_2_), cold storage (CS), yellow LED irradiation, and their combined application (N_2_+LED+CS) on the quality of Sichuan pepper during storage. While individual treatments have been reported, their combined effect remains unexplored; this approach simultaneously targets temperature, oxygen, and light. The research focused on how these methods influence qualitative characteristics, including the preservation of OH-α-sanshool (the numbing component), total polyphenol content (TPC), total flavonoid content (TFC), and flavor compounds over a 60-day storage period. Preliminary screening of individual storage factors (temperatures: 5, 10, 20, 40 °C; O_2_ levels: 0%, 10%, 20%, 40%; and LED colors: white, red, blue, yellow) showed that 5 °C and 0% O_2_ significantly slowed degradation of TPC, TFC, and flavor compounds, while yellow LED notably preserved OH-α-sanshool. The combined N_2_+LED+CS approach was particularly effective, reducing losses in TPC and TFC by 46.88% and 57.00%, respectively, compared to the control group. Additionally, OH-α-sanshool levels under N_2_+LED+CS conditions were maintained at 19.04 mg/g, representing a 115.38% reduction in loss. The study concludes that the N_2_+LED+CS method is a simple and efficient strategy that does not rely on chemical preservatives, offering a potentially sustainable approach to Sichuan pepper preservation.

## 1. Introduction

Sichuan pepper (*Zanthoxylum bungeanum* Maxim), also called huajiao, belongs to the rind of the pepper plant in the Rutaceae family, and can be categorized into “green Sichuan pepper” and “red Sichuan pepper” [1,2]. The world has about 250 species of Sichuan pepper plants, mainly in China, Korea and Japan, with about 45 species present in China [3]. Sichuan pepper is widely desired by consumers for its unique flavor and numbing sensation, which have been used as a seasoning spice for 2000 years in China [4]. A number of important chemical components, such as essential oil, polyphenols, and phenylpropanes, make up Sichuan pepper [5]. As an affinal drug and diet plant, Sichuan pepper is not only used for cooking, but can also be used as medicine, which has clearing heat, appetizing, antioxidant and anti-cancer effects [6]. However, Sichuan pepper is affected by environmental conditions during storage, resulting in loss of flavor, odor, active compounds (phenolic and flavonoid), and numbing compounds (OH-α-sanshool), which not only reduces the quality of pepper, but also causes significant losses to the economy. It is vital to do a thorough study aimed at reducing Sichuan pepper deterioration because of how crucial it is to maintain its flavor while being stored.

By altering the concentration of gases (O_2_, CO_2_, and N_2_) in the packaging environment, a modified atmosphere can delay plant ripening, cellular disintegration, stunt vitamin development, and lower enzyme activity, all of which help to minimize the quality loss of fruits and vegetables during storage [7,8]. The type and concentration of the gas inside the modified atmosphere packaging is dependent on the raw material of the food [9]. Moreover, modified atmosphere packaging has been recognized for consumers owing to the absence of synthetic chemicals and the absence of toxic residues on the product. In this context, Chen et al. [10] explored the influence of air-conditioned packaging on cabbage quality, lignin biosynthesis, reactive oxygen species metabolism and stem tissue structure. This finding showed that modified atmosphere packaging inhibited lignin synthesis, reactive oxygen species content and reduced the quality loss of cabbage during storage. In addition, modified atmosphere packaging can reduce the oxidative degradation of raw flavor compounds and during storage through adjusting O_2_ content [11]. Light, an important factor affecting plants, not only provides energy for photosynthesis but also signals physiological responses [12]. LED light sources provide inexpensive, efficient and controllable light sources that can selectively and quantitatively deliver different spectra compared to conventional light sources. Interestingly, Sichuan pepper is susceptible to light exposure during storage, which results in the conversion and degradation of OH-α-sanshool [13]. Recent studies have demonstrated that combining modified atmosphere packaging (MAP) with LED irradiation exerts synergistic effects on quality maintenance of fresh produce, such as okra [14] and fresh-cut carrots [15]. However, most of these investigations have focused on extending the shelf life or delaying the senescence of fresh fruits and vegetables, with limited attention given to the preservation of key quality components in dried spices. This is problematic because it is hard to preserve Sichuan pepper quality during preservation using a single component, given the complex environmental factors that affect it. LED and modified atmosphere packaging as combined storage methods are characterized by high safety, low energy consumption and high efficiency, which therefore merits further investigation.

The effects of N_2_, LED (yellow), CS, and N_2_+LED+CS approaches on Sichuan pepper quality during storage are examined in this paper. Antioxidant activity, TPC, and TFC were assessed for Sichuan pepper using various storage methods. Furthermore, electronic nose (e-nose) and headspace-gas chromatography-mass spectrometry (HS-GC-MS) were used to assess Sichuan pepper’s odor and flavor components under different storage conditions. Lastly, HPLC was used to investigate how different storage methods affected the amount of OH-α-sanshool. It was hypothesized that the combined N_2_+LED+CS treatment would exhibit a synergistic effect, preserving the quality of Sichuan pepper more effectively than any single treatment alone.

## 2. Materials and Methods

### 2.1. Materials

Sichuan pepper was supplied by Xuemailong Food Flavor Co. (Zhengzhou, China). Methanol (chromatographically pure) was obtained from Sigma-Aldrich Co. (Shanghai, China). OH-α-sanshool standard of > 95% purity (CAS: 83883-10-7) was obtained from Nanjing Bencao Yikang Biotechnology Co. (Nanjing, China). LED strip lights (white, wave peaks: 540 nm, red, wave peaks: 600 nm, blue, wave peaks: 440 nm, yellow, wave peaks: 580 nm) purchased from Rays Lighting Development Co. (Linyi, China). Polyethylene terephthalate (PET, 28 cm × 40 cm × 80 µm) adhesive pouches were obtained from Amisen New Material Technology Co. (Xiamen, China).

### 2.2. Storage Factor Analysis of Sichuan Pepper

The storage temperatures (5, 10, 20, 40 °C) were selected to cover typical refrigeration (5 °C), ambient (20–25 °C), and elevated (40 °C) storage conditions, allowing assessment of temperature effects across a wide range relevant to actual supply chains. The O_2_ concentrations (0%, 10%, 20%, 40%) were chosen to represent anaerobic (0%), low-oxygen (10%), atmospheric (20%), and high-oxygen (40%) environments, facilitating systematic evaluation of oxygen-dependent degradation. LED wavelengths (white: 540 nm, red: 600 nm, blue: 440 nm, yellow: 580 nm) were selected based on their distinct spectral properties and previous reports indicating their differential effects on plant bioactive compounds.

CK group: A total of 300 g of Sichuan pepper was placed in 50 × 50 cm × 50 cm polypropylene (PP) trays and spread evenly for storage at room temperature (25 ± 2 °C). During the 60-day storage period, samples were acquired on the 15th, 30th, 45th, and 60th days.

Temperature group: A total of 300 g of Sichuan pepper was placed in 50 × 50 cm × 50 cm PP trays, spread evenly and stored at different temperatures (5 °C, 10 °C, 20 °C, 40 °C). During the 60-day storage period, samples were acquired on the 15th, 30th, 45th, and 60th days.

LED group: A total of 300 g of Sichuan pepper was put into 50 × 50 cm × 50 cm PP trays and spread evenly. After that, the white, red, blue, and yellow LED light strips (of identical length and number of light-emitting units) were spread out equally over the cardboard and placed directly above the PP trays at a distance of approximately 30 cm from the sample surface, with a light intensity of 20 µmol·m^−2^·s^−1^, for Sichuan pepper irradiation storage at room temperature (25 ± 2 °C). The irradiation was applied continuously throughout the entire 60-day storage period to evaluate the effects of different light spectra. During the 60-day storage period, samples were acquired on the 15th, 30th, 45th, and 60th days.

O_2_ content group: A total of 100 g of Sichuan pepper was put into PET and filled with different ratios of O_2_ (0%, 10%, 20%, 40%) and N_2_ using a gas-conditioned packaging machine (LQ-370, Strong Force Packaging Technology Co., Ningbo, China). The PET pouches were evacuated for 5 s to remove residual air, then immediately filled with the preset gas mixture, and finally heat-sealed at approximately 150 °C to ensure complete airtightness. The sealing process was performed to achieve firm closure without damaging the pouch material. All packaged samples were visually inspected for sealing integrity before storage. The residual oxygen concentration was determined according to the gas-conditioned packaging machine’s programmed settings, which were calibrated and validated prior to use. The same procedure applies to the N_2_ and N_2_+LED+CS groups described below. Following encapsulation, Sichuan pepper was stored at room temperature (25 ± 2 °C). During the 60-day storage period, samples were acquired on the 15th, 30th, 45th, and 60th days.

### 2.3. Comparison of Different Storage Methods for Sichuan Pepper

CK group: To be stored at room temperature (25 ± 2 °C), the 300 g of Sichuan pepper was dispersed uniformly in 50 × 50 cm × 50 cm PP trays. During the 60-day storage period, samples were acquired on the 15th, 30th, 45th, and 60th days.

CS group: The 300 g of Sichuan pepper was placed in 50 cm × 50 cm × 50 cm PP trays, spread evenly and stored at 5 ± 1 °C. Samples were collected on the 15th, 30th, 45th, and 60th days of the 60-day storage period. During the 60-day storage period, samples were acquired on the 15th, 30th, 45th, and 60th days.

N_2_ group: The 100 g of Sichuan pepper was put into PET and filled with N_2_ (100%) using a gas-conditioned packaging machine (LQ-370, Strong Force Packaging Technology Co., Zhejiang, China). The PET was evacuated for 5 s and quickly filled with the set ratios of gases, and finally heat-sealed at approximately 150 °C to ensure complete airtightness. The sealing process was performed to achieve firm closure without damaging the pouch material. All packaged samples were visually inspected for sealing integrity before storage. After encapsulation, the Sichuan pepper was stored at a temperature of (5 ± 1 °C). During the 60-day storage period, samples were acquired on the 15th, 30th, 45th, and 60th days.

LED group: The 300 g of Sichuan pepper was put into 50 cm × 50 cm × 50 cm PP trays and spread evenly. Then the LED light strips (yellow) were evenly distributed on the cardboard and covered on the PP trays at a distance of approximately 30 cm from the sample surface, with a light intensity of 20 µmol·m^−2^·s^−1^, and irradiated continuously for irradiation storage of Sichuan pepper. During the 60-day storage period, samples were acquired on the 15th, 30th, 45th, and 60th days.

N_2_+LED+CS group: The 100 g of Sichuan pepper was put into PET and filled with N_2_ (100%) using a gas-conditioned packaging machine (LQ-370, Strong Force Packaging Technology Co., Ltd., Zhejiang, China). The PET was evacuated for 5 s and quickly filled with the set ratios of gases, and finally heat-sealed at approximately 150 °C to ensure complete airtightness. The sealing process was performed to achieve firm closure without damaging the pouch material. All packaged samples were visually inspected for sealing integrity before storage. Following encapsulation, the yellow LED light strips were equally spaced out on the PP trays at a distance of approximately 30 cm from the sample surface, with a light intensity of 20 µmol·m^−2^·s^−1^, and irradiated continuously for Sichuan pepper irradiation storage at 5 ± 1 °C. During the 60-day storage period, samples were acquired on the 15th, 30th, 45th, and 60th days.

### 2.4. Determination of Total Polyphenol Content

To initiate the extraction process, Sichuan pepper (10.0 g) was homogenized with 200 mL of 70% (*v*/*v*) ethanol solution. Ultrasonic-assisted extraction (500 W, 1 h) was subsequently performed to ensure complete dissolution of the material. Following extraction, phase separation was achieved by centrifugation (8000 rpm, 10 min, 25 °C), and the clarified supernatant was collected for subsequent analysis. The colorimetric method was used to perform the TPC [16]. Specifically, the supernatant aliquot (5 mL) was mixed with 0.2 M Folin–Ciocalteu reagent (5 mL). The mixture was incubated for 5 min before being combined with 6 mL of a 10%, m/v Na_2_CO_3_ solution. Following a 30 min equilibration period under ambient conditions, absorbance at 760 nm was measured using an ultraviolet spectrophotometer (UV1800, Shimadzu Corporation Co., Suzhou, China). A calibration curve constructed using gallic acid standards allowed the conversion of absorbance data to TPC (GAE/g).

### 2.5. Determination of Total Flavonoid Content

The extraction of TFC from Sichuan pepper was the same as in Section 2.4. The TFC was determined using a colorimetric method [17]. The supernatant (1 mL) was mixed with NaNO_2_ (2 mL, 10%, m/v) over 5 min. Then, AlCl_3_∙6H_2_O (2 mL, 10%, m/v) was added to the mixed solution over 6 min. After completion of the reaction, NaOH solution (2 mL, 20%, m/v,) was added over 5 min. The absorbance value at 510 nm was determined using an ultraviolet spectrophotometer. A calibration curve constructed using rutin standards allowed the conversion of absorbance data to TFC (mg RE/g).

### 2.6. Determination of Antioxidant Activity

A volume of 20 mL of 70% (*v*/*v*) aqueous ethanol was used to extract 1 g of Sichuan pepper, which was then ultrasonically assisted (500 W) for one hour. After extraction, the mixture was centrifuged in a chilled centrifuge (8000 rpm, 10 min, 25 °C). The resultant supernatant was filtered through a 0.22 μm organic membrane and stored at 5 °C.

#### 2.6.1. Determination of Scavenging Rate of DPPH

The scavenging rate of DPPH radicals was referred to Shen et al. [18]. In short, the 300 μL supernatant was reacted for 30 min with DPPH (2 mL, 0.42 mM) in a light-avoidance setting. Following the completion of the reaction, an ultraviolet spectrophotometer was used to measure the reacted mixed solution’s absorbance value at 517 nm. The formula is shown below:

(1) Free radical scavenging rate = 100% × (B_0_ − (B − B_1_))/B_0_

where B_0_ denotes the absorbance value of the DPPH solution, B denotes the absorbance value of extract after reaction with DPPH, and B_1_ denotes the absorbance value of the extract.

#### 2.6.2. Determination of Scavenging Rate of ABTS

The method described by Lee et al. [19] was employed to assess the scavenging rate of sample for ABTS radical. Initially, K_2_S_2_O_8_ (2.45 mM) and ABTS solution (7 mM) were reacted for 12 h at room temperature and shielded from light to produce ABTS^+^ solution. First, the ABTS^+^ solution should be diluted to an absorbance value of 0.700 ± 0.02 at 734 nm. After that, the supernatant (1 mL) and 2 mL of ABTS^+^ were combined for 6 min. The absorbance value was assessed using a UV spectrophotometer. Using Equation (1) above, the Sichuan pepper’ rate of scavenging ABTS radicals during storage was determined.

### 2.7. E-Nose Analysis

To fully assess the odor, Sichuan pepper (1 g) was first allowed to equilibrate in a 20 mL airtight vial. The entire testing period was 120 s, and the headspace gas flow rate was 1.0 L/min. Following sample testing, the apparatus was cleaned for 120 s at a flow rate of 1.0 L/min, and then further tests were conducted.

### 2.8. HS-GC-MS for the Analysis of Flavor Compounds

With minor modifications, the approach described by Shen et al. [18] served as the basis for determining the flavor components of Sichuan pepper. HS-GC-MS (Agilent 8890-5977B, Agilent Technologies, Santa Clara, CA, USA) fitted with an HP-5MS fused silica capillary column (30 mm × 0.25 mm, 0.25 µm) was utilized to identify the flavor components of Sichuan pepper. The Sichuan pepper (1 g) was incubated for 15 min at 50 °C. It took 5 min to extract the Sichuan pepper’s flavor ingredients. The helium was employed as the carrier gas (flow rate: 1 mL/min). The heating program was as follows: from 50 °C to 70 °C (rate: 2 °C/min), from 70 °C to 120 °C (rate: 5 °C/min), and from 120 °C to 250 °C (rate: 10 °C/min) and held for 3 min. An internal reference for the flavor compounds of Sichuan pepper was 2,4,6-trimethylpyridine (0.1 mg/g). Under the aforementioned circumstances, a mixture of C7–C30 alkanes was analyzed, and the compounds’ retention index (RI) values were determined. Mass spectrometry (>85% match) and RI were used to identify Sichuan pepper flavor components.

### 2.9. HPLC Analysis of OH-α-Sanshool Content

The extraction method of OH-α-sanshool from Sichuan pepper was the same as in Section 2.6. Using a C18 column (Thermo fisher, Shanghai, China, 4.6 mm × 75 mm, 5 μm) and a high-performance liquid chromatography (HPLC) system (e2695, Waters, Milford, CT, USA), the variations in OH-α-sanshool concentration over storage were assessed. The column temperature and injection volume were 30 °C and 20 µL respectively. Ultrapure water (A) and methanol (B) served as the HPLC’s mobile phases (rate: 0.8 mL/min). The ultraviolet absorption wavelength was set to 270 nm for the determination of OH-α-sanshool. The gradient elution procedure was as follows: 50~70% B was used at 0~15 min, 70% B was used at 15~25 min, and 70~50% B was used at 25~30 min.

### 2.10. Data Analysis

This study’s data is presented as mean ± standard deviation, with three independent biological replicates (separate samplings) for each treatment and sampling time point. For each biological replicate, the sample was thoroughly homogenized and measured in triplicate (technical replicates), and the mean values were used for subsequent data analysis. SPSS (version 23.0) was used for statistical analysis by one-way analysis of variance (ANOVA) and Dunkan’s tests (*p* < 0.05). Prior to ANOVA, normality and homogeneity of variances were assumed based on the experimental design and preliminary data inspection.

## 3. Results and Discussion

### 3.1. Effect of Different Factors on TPC and TFC of Sichuan Pepper

**Figure 1:**
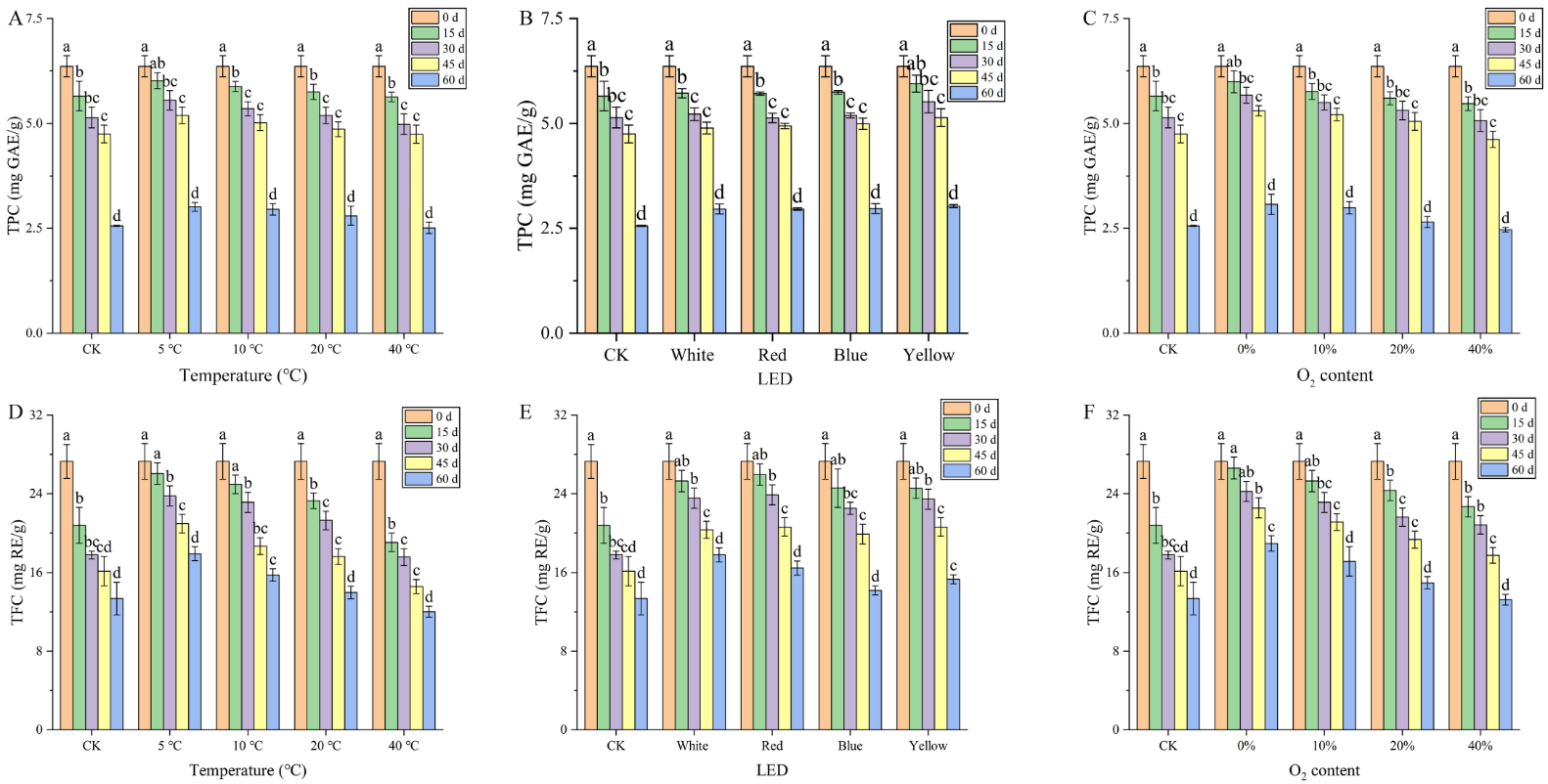
Variations in TPC (**A**–**C**) and TFC (**D**–**F**) of Sichuan pepper depending on different factors during storage. Note: Lowercase letters represent significant differences within groups.

Sichuan pepper has potent antibacterial, anti-inflammatory, and antioxidant properties due to its abundance of polyphenols and flavonoid active components. Additionally, polyphenols and flavonoids have a significant impact on Sichuan pepper’s color sensory indices. The effects of temperature, LED, and O_2_ content on Sichuan pepper’s TPC and TFC during storage were examined, as illustrated in Figure 1. Over time, Sichuan pepper’s TPC and TFC dropped as storage duration increased. Before the storage experiment began, the TPC and TFC were 6.36 GAE/g and 27.29 mg RE/g, respectively. On days 15, 30, 45, and 60 of storage, the TPC in the CK group was 5.65 GAE/g, 5.14 GAE/g, 4.75 GAE/g, and 2.56 GAE/g, respectively. On the 15th, 30th, 45th, and 60th days, the TFC was 20.79 mg RE/g, 17.78 mg RE/g, 16.13 mg RE/g, and 13.35 mg RE/g. Consequently, following 60 days, the TPC and TFC dropped by 59.75% and 51.08%, respectively. The TPC of Sichuan pepper stored at 5 °C, 10 °C, 20 °C, and 40 °C was 3.01 GAE/g, 2.95 GAE/g, 2.80 GAE/g, and 2.51 GAE/g after 60 days in the temperature group; TFCs were 17.90 mg RE/g, 15.74 mg RE/g, 13.96 mg RE/g, and 12.01 mg RE/g (Figure 1A,D). Sichuan pepper stored in the LED group under white, red, blue, and yellow LED storage conditions had TPCs of 2.96 GAE/g, 2.95 GAE/g, 2.97 GAE/g, and 3.03 GAE/g after 60 days. The TFC content was 16.80 mg RE/g, 16.45 mg RE/g, 14.15 mg RE/g, and 15.29 mg RE/g (Figure 1B,E). Sichuan pepper held in the O_2_ content group for 60 days had a TPC of 3.07 GAE/g, 2.99 GAE/g, 2.65 GAE/g, and 2.47 GAE/g under storage conditions of 0%, 10%, 20%, and 40% O_2_ content, and TFCs of 18.96 mg RE/g, 17.13 mg RE/g, 14.96 mg RE/g, and 13.23 mg RE/g (Figure 1C,F). After 60 d of storage at 5 °C, the loss of TPC and TFC was reduced by 17.58% and 34.08%, respectively, compared with the CK group. However, after 60 days at 0% O_2_ content, the loss of TPC and TFC was reduced by 19.92% and 42.00%, respectively. There was no statistically significant effect of LED on TPC and TFC of Sichuan pepper during the storage period. These findings demonstrate that storage conditions with low temperatures and a low O_2_ concentration are better for improving TPC and TFC retention in Sichuan pepper over time. According to Ivane et al. [20], the primary polyphenols and flavonoids in Sichuan pepper are epicatechin, quercetin, catechin, and rutin. It is well recognized that flavonoids and polyphenols have potent antioxidant properties [21]. Consequently, reducing the O_2_ level while storing Sichuan pepper can lessen the amount of TPC and TFC that is lost over time. Additionally, low temperatures also considerably decreased the loss of Sichuan pepper in storage TPC and TFC since high temperatures encourage the oxidation and degradation of polyphenols [22]. The application of LED in fruit and vegetable preservation increases TPC and TFC, a phenomenon in which LED stimulates the up-regulation of a number of genes involved in the expression of specific gene-encoded enzymes (such as chorismate mutase, 3-deoxy-D-arabin-heplosonate 7-phosphate synthase, phenylalanine deaminase, and anthraquinone synthase) promoting polyphenol and flavonoid substance overexpression [14]. However, it is worth noting that in this experiment, Sichuan pepper was a dried pepper that had undergone high-temperature drying, and thus LED had no significant effect on TPC and TFC during storage. The protective effects of low temperature and oxygen restriction are mechanistically straightforward, as they directly suppress oxidative reaction kinetics and substrate availability [22]. In contrast, the beneficial effects of LED on phenolic and flavonoid accumulation in fresh produce rely on the up-regulation of biosynthesis-related genes and enzymes [14]. In dried Sichuan pepper, however, such light-responsive biosynthetic pathways are largely inactivated due to high-temperature processing, which likely explains the absence of a significant LED effect. In conclusion, Sichuan pepper’s TPC and TFC were negatively impacted by storage conditions with low temperatures and a low oxygen concentration.

### 3.2. Effect of Different Factors on Odor and Flavor of Sichuan Pepper

**Figure 2:**
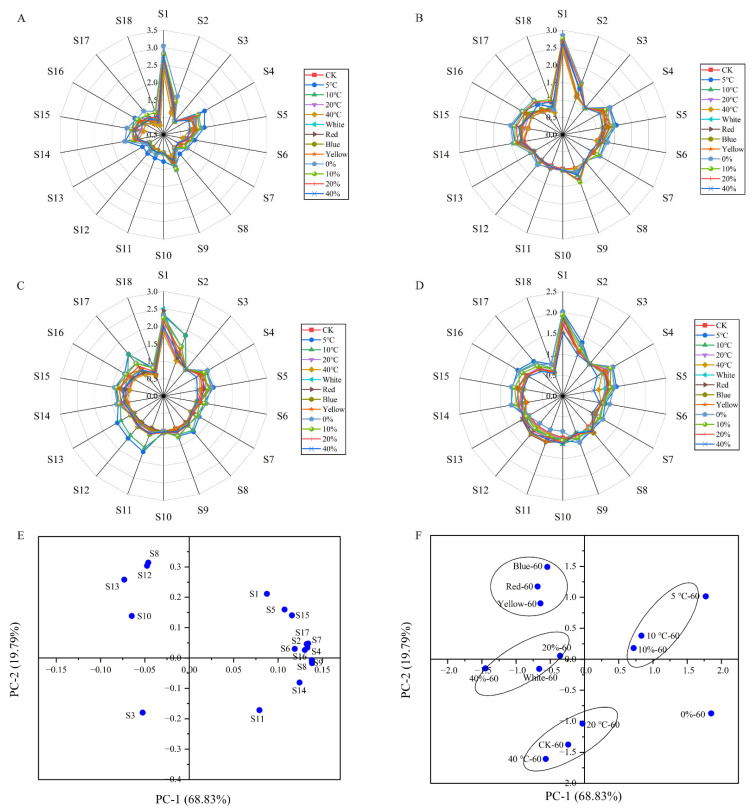
Odor analysis of Sichuan pepper under different storage conditions using e-nose during storage ((**A**), 15th day, (**B**), 30th day, (**C**), 45th day, (**D**), 60th day); Principal component analysis (loading (**E**) and scores (**F**) plots) of e-nose data of Sichuan pepper under different storage conditions. Note: Lowercase letters represent significant differences within groups. S1 (alkanes, smoke), S2, (alcohols, aldehydes, short-chain alkanes), S3 (ozone), S4 (sulphides), S5 (organic amines), S6 (organic gases, benzene, alcohols, aldehydes, ketones, aromatic compounds), S7 (short-chain alkanes), S8 (short-chain alkanes), S9 (aromatic compounds, alcohols, aldehydes), S10 (hydrogen), S11 (alkanes, olefins), S12 (short-chain alkanes), S13 (methane), S14 (combustible gases), S15 (organic gases), S16 (sulphides), S17 (nitriles) and S18 (ketones and alcohols).

Flavor and odor are the main determining variables in evaluating the qualities of Sichuan pepper. The odor variation of Sichuan pepper was evaluated using e-nose, a quick and non-destructive detecting technique. The e-nose is equipped with 18 sensors [23]. Radar plots of Sichuan pepper response signals to 18 sensors at the 15th, 30th, 45th, and 60th day of storage under various storage factor circumstances are displayed in Figure 2A–D. The findings demonstrate that, with the exception of S3 and S10, the response values of the S1 through S18 sensors exhibited a declining trend while Sichuan pepper was stored under various factor circumstances. This result indicates that the odor of Sichuan pepper decreases under different factors of storage conditions. However, the odor of Sichuan pepper did not contain S3 or S10 during storage, which is explained by the fact that Sichuan pepper does not contain ozone and hydrogen [24]. Meanwhile, the CK group exhibited the lowest Sichuan pepper odor response value during storage, while the greatest signal levels were recorded under 5 °C and 0% O_2_ content. In the meantime, LED had no discernible impact on Sichuan pepper’s odor while it was being stored. These findings show that Sichuan pepper odor loss during storage can be considerably minimized by using low temperature and low O_2_ content storage methods.

As shown in Figure 2E,F, principal component analysis (PCA) was used to examine the impact of different types of storage methods on the odor of Sichuan pepper. Two primary variables, PC-1 and PC-2, were obtained for PCA and accounted for 88.16% of the variance. The respective contributions of PC-1 and PC-2 were 72.53% and 15.63%. Four major categories can be used to classify the effects of various storage variables on Sichuan pepper’s odor over an extended period. The distances of 5 °C, 10 °C, 0% O_2_ content and 10% O_2_ content are on the rightmost side, indicating that Sichuan pepper stored at 5 °C, 10 °C, 0% O_2_ content and 10% O_2_ content maintains its odor better during the storage process. This separation along PC-1 largely reflected differences in the major odor-active volatiles, namely linalool, D-limonene, β-myrcene, and linalyl acetate. Samples stored at 5 °C and 0% O_2_ fell closest to fresh pepper (CK0), indicating better retention of these compounds, whereas the 60-day CK group deviated markedly, showing extensive volatile loss. The E-nose data corroborated the GC-MS findings, both indicating that low temperature and O_2_ restriction effectively preserved aroma, with LED exerting little effect on the volatile profile. In conclusion, Sichuan pepper’s odor loss during storage can be minimized by using low-temperature and low-oxygen-content storage methods.

Although e-nose is a valuable tool for quickly evaluating food odor, it is unable to detect changes in flavor chemicals that are present in food products over time [25]. HS-GC-MS was used to determine the identification and content changes in flavor compounds in Sichuan pepper from CK, temperature, LED and O_2_ content groups. Sichuan pepper was found to contain a total of 18 flavor components (Table 1). The main flavoring components of Sichuan pepper before the storage tests were linalool (32.24 μg/g), D-limonene (10.24 μg/g), β-Myrcene (2.70 μg/g), and linalyl acetate (2.36 μg/g). Zhang et al. [26] used GC-MS to identify the flavor components of fresh and dried Sichuan pepper. The findings showed that the main flavoring components in each sample were β-laurene, linalool, and D-limonene. Meanwhile, GC-MS was used to identify the flavor compounds of ultrasound-extracted Sichuan pepper volatile oil, and similar conclusions were obtained [27]. The flavor compounds of Sichuan pepper declined significantly after 60 days of storage under different storage factors. In CK60, the contents of linalool, D-limonene, β-Myrcene and linalyl acetate, the main flavor compounds of Sichuan pepper, were 18.37 μg/g, 4.51 μg/g, 1.01 μg/g and 1.17 μg/g, respectively. Meanwhile, the loss of flavor compounds of Sichuan pepper can be effectively reduced under low-temperature and low-O_2_-content storage conditions. The contents of linalool, D-limonene, β-Myrcene and linalyl acetate in Sichuan pepper were 27.33 μg/g, 7.38 μg/g, 1.85 μg/g, and 1.95 μg/g during storage at 5 °C. Meanwhile, at 0% O_2_ content, the main flavor compound contents of Sichuan pepper, linalool, D-limonene, β-Myrcene and linalyl acetate, were 26.56 μg/g, 8.10 μg/g, 2.03 μg/g, and 1.91 μg/g. The flavor compounds of Sichuan pepper did not undergo significant changes under different LED storage conditions, which is consistent with the TPC and TFC results. The CK group lost a lot of flavor components during storage since these chemicals are very volatile and oxidative [28]. In conclusion, Sichuan pepper’s flavor ingredients can be preserved longer by using methods of preservation that involve low temperatures and a low oxygen concentration.

### 3.3. Effect of Different Factors on OH-α-Sanshool of Sichuan Pepper

The numbing substances of Sichuan pepper are mainly amide compounds (OH-α-sanshool, OH-β-sanshool) [29]. As indicated in Table 2, the total amount of OH-α-sanshool in the CK group dropped by 76.20%, from 37.15 mg/g on the 0th day to 8.84 mg/g. The level of OH-α-sanshool was 11.19 mg/g and 9.25 mg/g at 5 °C and 10 °C, respectively. This suggests that low temperatures are advantageous for minimizing the loss of OH-α-sanshool. However, the amount of OH-α-sanshool was 7.01 mg/g over 60 days of storage at 40 °C, indicating that OH-α-sanshool is sensitive to temperature changes. This phenomenon may be attributed to the promotion of OH-α-sanshool degradation under high-temperature conditions [18]. Under yellow LED storage conditions, the LED group’s maximum retention of OH-α-sanshool was 11.34 mg/g. Photostimulation causes OH-α-sanshool to isomerize into OH-β-sanshool when exposed to light. The yellow LED can avoid the light absorption peak of OH-α-sanshool with the provision of low energy wavelengths, thus reducing the occurrence of photo-stimulation and subsequent degradation reactions [15]. After 60 days of storage at different O_2_ contents, the results showed that the content of OH-α-sanshool was 15.14 mg/g and 14.21 mg/g at 0% O_2_ content and 10% O_2_ content. On the other hand, OH-α-sanshool’s level was 7.38 mg/g after 60 days of storage at 40% O_2_ content, indicating that it is sensitive to the effects of O_2_. Unsaturated alkyl groups are very prone to oxidation to hydroxyls, ketones, or aldehydes, which leads to decline [30]. Overall, low temperature and a low O_2_ content can effectively retain OH-α-sanshool loss during storage. OH-α-sanshool contains an unsaturated amide structure and a conjugated diene system, making it susceptible to thermal, oxidative, and photochemical degradation. Elevated temperatures promote both thermal isomerization to OH-β-sanshool and oxidative cleavage of the unsaturated alkyl chain. Oxygen exposure facilitates oxidative degradation through the formation of hydroxylated and carbonyl derivatives [30]. Light irradiation, in turn, induces photoisomerization via excitation of the conjugated diene system. Beyond these chemical pathways, preserving OH-α-sanshool is critical for sensory quality and commercial value, as it is the primary contributor to Sichuan pepper’s characteristic numbing sensation [31].

### 3.4. Effect of N_2_+LED+CS on TPC and TFC of Sichuan Pepper

**Figure 3:**
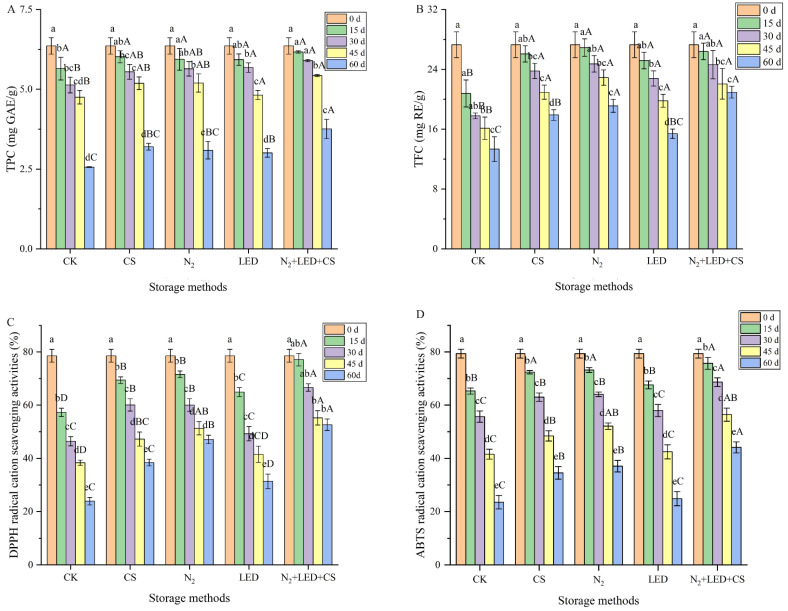
TPC (**A**), TFC (**B**) and antioxidant activity ((**C**), DPPH radical scavenging rate, (**D**), ABTS radical scavenging rate) of Sichuan pepper under storage method. Note: Uppercase letters indicate significant differences between groups; lowercase letters represent significant differences within groups.

Based on the screening of conditions in the above experiments, it was found that low temperature, low O_2_ content and yellow LED could minimize Sichuan pepper’s quality degradation while being stored. The effects of several methods of storage (CK, CS, N_2_, LED, and N_2_+LED+CS) on the TPC and TFC of Sichuan pepper were examined, as illustrated in Figure 3A,B. On the 0th day, the TPC and TFC in the CK group were 6.36 mg GAE/g and 27.29 mg RE/g. On the 15th, 30th, 45th, and 60th days of storage, the TPC of Sichuan pepper was 5.65 mg GAE/g, 5.14 mg GAE/g, 4.75 mg GAE/g, and 2.56 mg GAE/g, respectively. On the 15th, 30th, 45th, and 60th days, the TFC was 20.79 mg RE/g, 17.78 mg RE/g, 16.13 mg RE/g, and 13.35 mg RE/g. The TPC of Sichuan pepper stored using the CS, N_2_, LED, and N_2_+LED+CS methods was 3.01 mg GAE/g, 3.09 mg GAE/g, 3.10 mg GAE/g, and 3.76 mg GAE/g after 60 days of storage. With CS, N_2_, LED, and N_2_+LED+CS methods, the TFC was 17.90 mg RE/g, 19.12 mg RE/g, 15.40 mg RE/g, and 20.96 mg RE/g, respectively. Comparing with CK group, TPC loss was reduced by 17.58%, 20.70%, 21.09% and 46.88% in CS, N_2_, LED and N_2_+LED+CS methods, respectively. Meanwhile, TFC loss was reduced by 34.08%, 43.22%, 15.36% and 57.00% for CS, N_2_, LED and N_2_+LED+CS methods, respectively. The findings demonstrate that the TFC and TPC losses of Sichuan pepper were considerably decreased over the storage period by the CS, N_2_, and N_2_+LED+CS techniques; the N_2_+LED+CS method was the most successful in lowering TFC and TPC losses. Because of their potent antioxidant properties, polyphenols and flavonoids can be preserved in Sichuan peppercorns by reducing the amount of oxygen during storage [32]. On the other hand, CS can successfully inhibit the oxidative degradation of polyphenols and flavonoids in Sichuan pepper during storage, because high temperatures can accelerate the breakdown of these compounds [17,33]. In conclusion, Sichuan pepper’s TPC and TFC loss during storage may be successfully minimized using the N_2_+LED+CS method.

### 3.5. Effect of N_2_+LED+CS on Antioxidant Activity of Sichuan Pepper

The impact of CS, N_2_, LED, and N_2_+LED+CS methods on the antioxidant activity of Sichuan pepper was examined and is illustrated in Figure 3C,D. The findings demonstrate that as storage time increased, Sichuan pepper’s antioxidant activity progressively declined. On the 0th, 15th, 30th, 45th, and 60th days, Sichuan pepper’s DPPH scavenging rate in the CK group was 78.37%, 69.42%, 60.08%, 47.25%, and 38.40%, respectively. ABTS radicals were scavenged at rates of 79.37%, 65.32%, 55.69%, 41.54%, and 23.53%. Following a 60-day storage period, Sichuan pepper’s DPPH radical scavenging for CS, N_2_, LED, and N_2_+LED+CS methods was 38.40%, 47.11%, 31.34%, and 52.64%, respectively. ABTS radical scavenging was 34.54%, 37.08%, 24.83%, and 44.13%. Meanwhile, the LED storage method had no significant effect on the antioxidant activity of Sichuan pepper. When compared to the CS and N_2_ storage methods, the N_2_+LED+CS approach considerably prevented the decrease in the antioxidant activity of Sichuan pepper. This outcome could be attributed to two factors. On the one hand, Sichuan pepper can reduce the degradation of active substances at low temperatures; on the other hand, storage under N_2_ conditions prevents the active compounds in Sichuan pepper from being oxygenated, which can further inhibit the active chemicals’ oxidative breakdown [18]. The observed changes in antioxidant activity closely paralleled the trends in TPC and TFC across all storage conditions. This correlation supports the view that phenolic and flavonoid compounds are the primary contributors to the radical-scavenging capacity of Sichuan pepper. The better retention of these bioactive constituents under CS, N_2_, and N_2_+LED+CS treatments thus directly accounted for the higher DPPH and ABTS scavenging rates observed in these groups.

### 3.6. Effect of N_2_+LED+CS on Odor and Flavor of Sichuan Pepper

**Figure 4:**
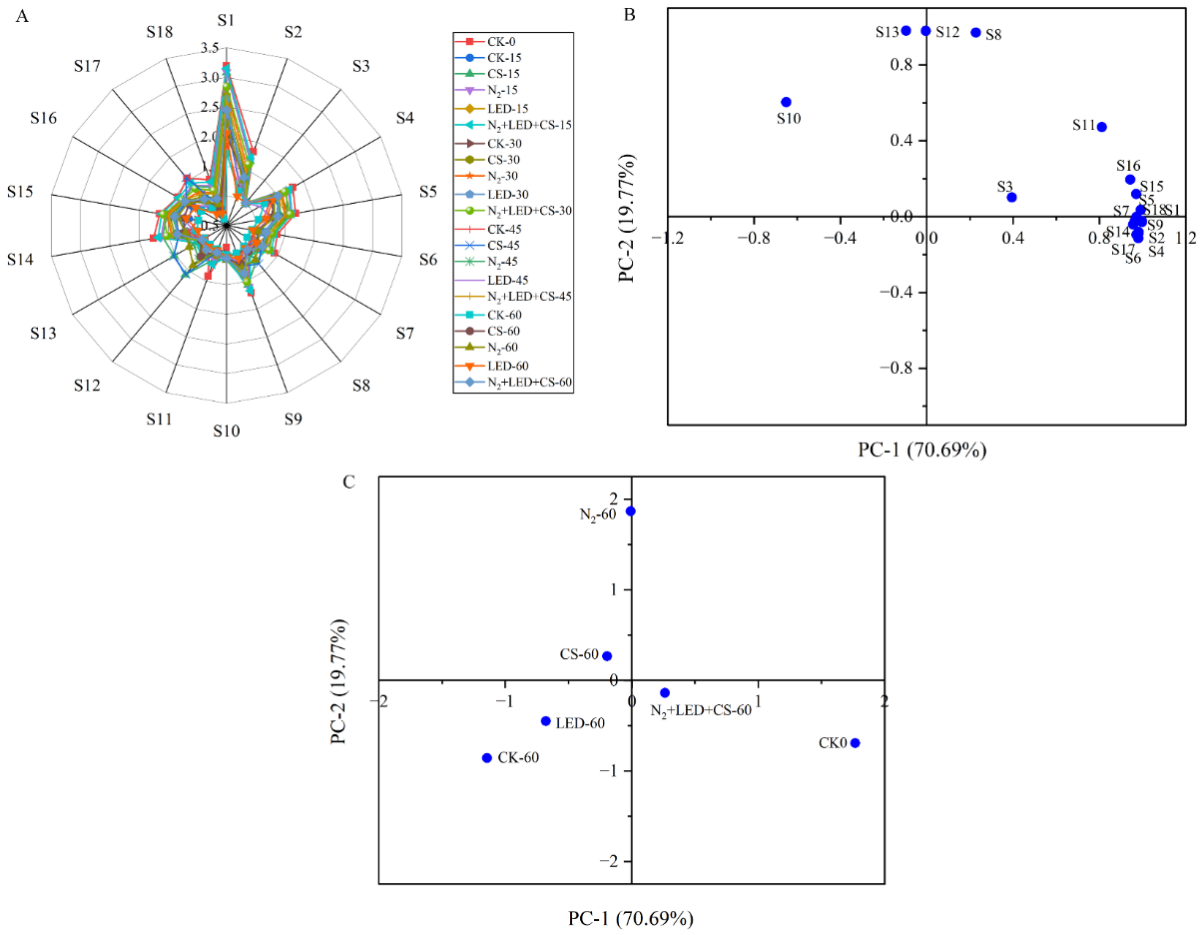
Odor analysis of Sichuan pepper from different storage methods using e-nose analysis during storage ((**A**), 60th day); Principal component analysis (loading (**B**) and scores (**C**) plots) of e-nose data of Sichuan pepper from different storage methods during storage.

The effects of CS, N_2_, LED, and N_2_+LED+CS methods on the odor of Sichuan pepper were investigated (Figure 4A). The radar plots show the response signals to 18 sensors during storage for the CS, N_2_, LED, and N_2_+LED+CS strategies. The findings demonstrate that during the storage period, the signal levels of the S1 to S18 of Sichuan pepper from various storage parties declined, with the exception of S3 (ozone) and S10 (hydrogen) sensors. Among all the storage methods, the CK group S1 to S18 had the minimum signal value, and the N_2_+LED+CS method had the maximum signal value during the storage period. According to these findings, Sichuan pepper’s odor loss during storage can be successfully minimized using the N_2_+LED+CS method. Principal component analysis was performed on e-nose data following 60 days of storage to investigate the impact of various storage techniques on the odor of Sichuan pepper. PCA produced two variables, PC-1 and PC-2, which together account for 90.46% of the variation, as seen in Figure 4B,C. PC-1 and PC-2 contributed 70.69% and 19.77%, respectively. This result demonstrates that the N_2_+LED+CS method could successfully minimize the odor loss of Sichuan pepper, as it was closer to CK0. Principal component analysis indicates that the efficiency of various methods of storage in lowering the odor loss of Sichuan pepper is ranked as: N_2_+LED+CS > N_2_ > CS > LED > CK.

The flavor compounds in Sichuan pepper were identified and determined using HS-GC-MS in accordance with various storage methods. This allowed researchers to examine how the flavor compounds in Sichuan pepper changed with time. The taste compound amounts of Sichuan pepper were compared across various preservation techniques, as indicated in Table 3. In line with the findings above, a total of 18 compounds were found in Sichuan pepper that were kept using various techniques. Linalool, D-limonene, β-Myrcene, and linalyl acetate were the main taste compounds found in Sichuan pepper, according to the results of the HS-GC-MS identification. At the end of storage, the flavor of the components in Sichuan pepper (linalool, 32.24 to 18.37 μg/g; D-limonene, 10.24 to 4.51 μg/g; and β-Myrcene, 2.70 to 0.21 μg/g) significantly decreased in the CK group. The contents of flavor compounds linalool in Sichuan pepper stored with CS, N_2_, LED and N_2_+LED+CS methods were 21.70 μg/g, 22.38 μg/g, 16.07 μg/g, and 24.93 μg/g, respectively. Meanwhile, the contents of D-limonene were 5.84 μg/g, 6.04 μg/g, 4.30 μg/g, and 6.51 μg/g. Among these storage methods, the N_2_+LED+CS method had a strong protective effect on the flavor compounds of Sichuan pepper, which reduced the linalool and D-limonene loss rate of Sichuan pepper by 35.71% and 44.35% during the storage period. There are two main explanations for this phenomenon. On the one hand, Sichuan pepper flavor components may not volatilize as much when stored using the CS method. The N_2_ storage method, on the other hand, can lessen the oxidative deterioration of the taste components in Sichuan pepper during storage because it possesses a low tolerance to O_2_ [18,23].

### 3.7. Effect of N_2_+LED+CS on OH-α-Sanshool of Sichuan Pepper

**Figure 5:**
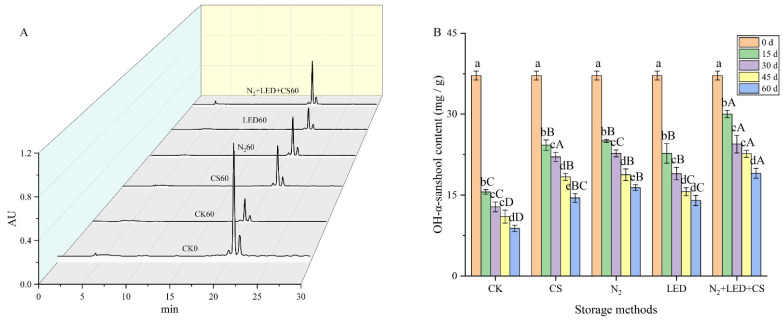
Variations in OH-α-sanshool content of Sichuan pepper of different storage methods during storage (chromatograms of OH-α-sanshool, (**A**); OH-α-sanshool content, (**B**)). Note: Uppercase letters indicate significant differences between groups; lowercase letters represent significant differences within groups.

Sichuan pepper from CS, N_2_, LED, and N_2_+LED+CS methods of storage underwent OH-α-sanshool content measurement using HPLC, as illustrated in Figure 5. The peak value at 28 min was OH-α-sanshool. Figure 5B displays the OH-α-sanshool content of Sichuan pepper stored using various methods of preservation throughout the period. The amount of OH-α-sanshool in the CK group dropped by 76.20%, from 37.15 mg/g to 8.84 mg/g (0~60 days). Sichuan pepper’s OH-α-sanshool loss during storage was considerably decreased by the CS, N_2_, LED, and N_2_+LED+CS methods of storage. The OH-α-sanshool levels of Sichuan pepper from the CS, N_2_, LED, and N_2_+LED+CS methods of storage were 14.46 mg/g, 16.39 mg/g, 14.01 mg/g, and 19.04 mg/g. Specifically, when compared to the CK group, the N_2_+LED+CS approach improved the retention of OH-α-sanshool by 115.38% over storage. When exposed to light, oxygen, and temperature, OH-α-sanshool becomes unstable and is prone to oxidation, degradation, and isomerization [30]. The alkyl group in OH-α-sanshool readily oxidizes to hydroxyl, ketones, or aldehydes due to the presence of unsaturated amides. Furthermore, OH-α-sanshool can be isomerized to OH-β-sanshool when exposed to light [13]. The superior performance of the combined N_2_+LED+CS treatment appears to stem from the complementary actions of its three components. CS retards the kinetics of chemical deterioration, including oxidative and thermal breakdown of polyphenols, flavonoids, and volatile flavor compounds [17,22]. N_2_ creates an oxygen-free environment, which mitigates oxidation of unsaturated alkyl groups in OH-α-sanshool and other labile flavor constituents [18,30]. Yellow LED, in turn, suppresses photo-induced isomerization of OH-α-sanshool by limiting exposure to photoactivating wavelengths, thereby reducing undesired photostimulation and subsequent degradation [13]. By simultaneously tackling the three primary degradation drivers—temperature, oxygen, and light—the combined strategy effectively outperforms any single treatment alone. The N_2_+LED+CS strategy retained 19.04 mg/g OH-α-sanshool after 60 days, well above the reported recognition threshold for *Z. bungeanum* pungency [31], indicating the effective preservation of the numbing sensation. Vacuum packaging alone, though beneficial for bioactive compound retention, does not specifically mitigate photo- or thermo-induced degradation [34]. The combined method thus provides a complementary solution by concurrently addressing oxidative, thermal, and light-driven quality loss without chemical additives. In conclusion, temperature, light, and O_2_ content have a greater impact on Sichuan pepper quality during storage than any one other aspect. Therefore, the storage method of N_2_+LED+CS can effectively protect the quality of Sichuan pepper in the storage period.

## 4. Conclusions

This study evaluated the effects of temperature, LED, and O_2_ content on Sichuan pepper quality. The condition screening showed that storage techniques with low temperatures and low oxygen content might lessen the loss of Sichuan pepper’s active ingredients, flavorings, and odors. Meanwhile, Sichuan pepper’s loss of numbing agents (OH-α-sanshool) can be minimized by using yellow LED, low temperatures, and low-oxygen-content storage methods. These findings imply that the quality of Sichuan pepper is influenced by multiple factors when it is being stored. As a result, the quality of Sichuan pepper in the storage period was examined using the CS, N_2_, LED, and N_2_+LED+CS methods. Of these, the N_2_+LED+CS method reduced the amount of Sichuan pepper’s active ingredients (TPC and TFC), as well as its flavor and odor while being stored. Additionally, the N_2_+LED+CS strategy considerably reduced the loss of OH-α-sanshool content and the deterioration of antioxidant activity in Sichuan pepper over time. Finally, as Sichuan pepper quality is affected by several conditions, the N_2_+LED+CS combination storage method may be able to reduce the amount of quality loss that occurs in storage, offering a promising non-chemical alternative to conventional preservation methods. The method is especially relevant for dried Sichuan pepper during storage and transit, and may also apply to other dried plant products prone to oxidative or light-induced deterioration. Future studies should evaluate the economic feasibility and operational complexity of this strategy in real industrial settings, including equipment investment, energy costs, and process integration into existing packaging lines.

## Figures and Tables

**Table 1:** Effects of storage factor on the flavor compound content of Sichuan pepper during storage.

Time (min)	RI	Compounds	Content (μg/g)													
					Temperature				LED				O_2_ Content			
			CK0	CK60	5 °C	10 °C	20 °C	40 °C	White	Red	Blue	Yellow	0%	10%	20%	40%
6.60	97	β-Pinene	0.76	0.21	0.56	0.39	0.27	0.20	0.49	0.39	0.38	0.46	0.61	0.45	0.42	0.25
8.41	97	β-Myrcene	2.70	1.01	1.85	1.50	1.16	0.99	1.50	1.20	1.18	1.35	2.03	1.47	1.34	1.14
8.90	92	2-Carene	0.35	0.14	0.26	0.23	0.19	0.13	0.23	0.18	0.17	0.23	0.28	0.21	0.23	0.18
9.68	97	D-Limonene	10.24	4.51	7.38	6.23	5.52	4.42	6.62	5.30	5.26	5.85	8.10	5.82	6.10	4.89
9.97	94	β-Phellandrene	1.39	0.53	1.10	0.77	0.67	0.52	0.91	0.73	0.71	0.83	1.10	0.80	0.72	0.60
10.25	97	Eucalyptol	1.48	0.07	0.11	0.10	0.09	0.07	0.92	0.73	0.71	0.83	1.17	0.86	0.12	0.10
11.45	97	Gamma-Terpinene	0.83	0.22	0.6	0.36	0.31	0.22	0.51	0.41	0.39	0.48	0.67	0.49	0.36	0.29
11.72	97	β-Ocimene	0.44	0.13	0.34	0.22	0.19	0.13	0.29	0.23	0.22	0.29	0.35	0.27	0.23	0.18
17.46	98	Thujone	1.14	0.48	0.91	0.76	0.64	0.47	0.81	0.65	0.63	0.74	0.93	0.68	0.67	0.51
18.78	94	Ethyl 2-(5-methyl-5-vinyltetrahydrofuran-2-yl)propan-2-yl carbonate	0.73	0.29	0.61	0.49	0.35	0.29	0.52	0.41	0.40	0.48	0.6	0.45	0.40	0.34
19.87	95	Pentadecane	0.10	0.02	0.05	0.04	0.03	0.02	0.05	0.04	0.03	0.08	0.08	0.07	0.06	0.06
21.05	87	Linalool	32.24	18.37	27.33	24.22	21.31	18.02	18.98	18.38	18.28	18.65	26.56	23.45	21.47	18.41
21.21	91	Linalyl acetate	2.36	1.17	1.95	1.42	1.22	1.15	1.68	1.34	1.32	1.51	1.91	1.38	1.17	1.13
21.96	99	Caryophyllene	1.21	0.52	0.87	0.79	0.73	0.51	0.74	0.59	0.57	0.68	0.98	0.72	0.73	0.59
22.37	96	Terpinen-4-ol	0.23	0.09	0.18	0.14	0.12	0.09	0.15	0.12	0.11	0.17	0.18	0.14	0.15	0.11
23.45	97	Humulene	0.52	0.17	0.35	0.31	0.23	0.17	0.33	0.26	0.25	0.32	0.41	0.31	0.28	0.19
23.82	93	Germacrene D	0.70	0.27	0.49	0.44	0.36	0.26	0.42	0.33	0.32	0.40	0.55	0.41	0.40	0.31
24.08	98	2-Cyclohexen-1-one, 3-methyl-6-(1-methylethyl)	0.07	0.04	0.07	0.06	0.05	0.03	0.06	0.04	0.03	0.08	0.06	0.06	0.08	0.04

Note: Changes in flavor compound contents of Sichuan pepper after 60 days of storage with different factors.

**Table 2:** Variations in OH-α-sanshool content of Sichuan pepper under different storage conditions.

Treatment		OH-α-Sanshool Content (mg/g)				
		0 d	15 d	30 d	45 d	60 d
	CK	37.15 ± 0.82 a	15.57 ± 0.42 b	12.81 ± 0.92 c	11.02 ± 1.21 c	8.84 ±0.58 d
Temperature (°C)	5 °C	37.15 ± 0.82 a	24.26 ± 0.96 b	22.09 ± 0.85 c	18.37 ± 0.63 d	14.46 ± 0.79 e
	10 °C	37.15 ± 0.82 a	20.19 ± 0.79 b	18.20 ± 0.84 c	15.12 ± 0.95 d	11.19 ± 0.56 e
	20 °C	37.15 ± 0.82 a	17.26 ± 0.85 b	15.57 ± 0.42 b	13.10 ± 0.92 c	9.25 ± 0.80 d
	40 °C	37.15 ± 0.82 a	15.12 ± 0.96 b	13.64 ± 0.61 bc	11.89 ± 0.52 c	7.01 ± 0.55 d
LED	White	37.15 ± 0.82 a	16.51 ± 0.19 b	14.77 ± 0.46 c	13.18 ± 0.31 d	8.35 ± 0.44 e
	Red	37.15 ± 0.82 a	14.20 ± 0.65 b	12.45 ± 0.39 c	11.24 ± 0.41 c	5.97 ± 0.46 d
	Blue	37.15 ± 0.82 a	15.04 ± 0.72 b	13.62 ± 0.38 bc	12.40 ± 0.56 c	5.35 ± 0.50 d
	Yellow	37.15 ± 0.82 a	19.38 ± 1.13 b	18.04 ± 0.39 bc	16.26 ± 0.74 c	11.34 ± 0.83 d
O_2_ content (%)	0	37.15 ± 0.82 a	24.60 ± 0.99 b	22.05 ± 0.88 c	18.59 ± 0.84 d	15.14 ± 0.65 e
	10	37.15 ± 0.82 a	21.54 ± 0.64 b	18.86 ± 0.77 c	16.16 ± 0.69 d	14.21 ± 1.23 d
	20	37.15 ± 0.82 a	20.74 ± 0.93 b	17.41 ± 0.95 c	15.01 ± 0.48 d	11.43 ± 0.95 e
	40	37.15 ± 0.82 a	17.39 ± 0.92 b	15.58 ± 0.46 b	13.34 ± 0.92 c	7.38 ± 0.72 d

Note: Data are presented as mean ± standard deviation (*n* = 3). Different lowercase letters (a–e) in the same row indicate significant differences among storage times (0, 15, 30, 45, 60 d) within the same treatment, as determined by one-way ANOVA followed by Duncan’s test (*p* < 0.05).

**Table 3:** Comparison of different storage methods on the flavor compound contents of Sichuan pepper.

Time (min)	RI	Compound	CK (μg/g)					CS (μg/g)				N2 (μg/g)			
			0 d	15 d	30 d	45 d	60 d	15 d	30 d	45 d	60 d	15 d	30 d	45 d	60 d
6.60	97	β-Pinene	0.76	0.56	0.39	0.27	0.21	0.56	0.53	0.50	0.32	0.62	0.57	0.57	0.33
8.41	97	β-Myrcene	2.70	1.85	1.50	1.16	1.01	1.85	1.77	1.64	1.22	2.18	1.88	1.73	1.36
8.90	92	2-Carene	0.35	0.26	0.23	0.19	0.14	0.26	0.26	0.24	0.18	0.31	0.27	0.25	0.19
9.68	97	D-Limonene	10.24	7.38	6.23	5.52	4.51	7.38	7.08	7.04	5.84	8.76	7.43	7.29	6.04
9.97	94	β-Phellandrene	1.39	1.10	0.77	0.67	0.53	1.10	0.99	0.84	0.67	1.16	1.05	0.87	0.69
10.25	97	Eucalyptol	1.48	0.11	0.10	0.09	0.07	0.11	0.11	0.10	0.08	1.23	0.11	0.10	0.08
11.45	97	Gamma-Terpinene	0.83	0.60	0.36	0.31	0.22	0.60	0.53	0.44	0.32	0.74	0.55	0.46	0.33
11.72	97	β-Ocimene	0.44	0.34	0.22	0.19	0.13	0.34	0.3	0.25	0.20	0.39	0.31	0.26	0.21
17.46	98	Thujone	1.14	0.91	0.76	0.64	0.48	0.91	0.86	0.81	0.60	1.03	0.91	0.84	0.62
18.78	94	Ethyl 2-(5-methyl-5-vinyltetrahydrofuran-2-yl)propan-2-yl carbonate	0.73	0.61	0.49	0.35	0.29	0.61	0.53	0.48	0.40	0.68	0.56	0.50	0.41
19.87	95	Pentadecane	0.10	0.05	0.04	0.03	0.02	0.05	0.05	0.04	0.03	0.08	0.06	0.04	0.03
21.05	87	Linalool	32.24	27.33	24.22	21.31	18.37	27.13	23.73	22.12	21.70	28.95	26.48	24.81	22.38
21.21	91	Linalyl acetate	2.36	1.95	1.42	1.22	1.17	1.95	1.74	1.61	1.25	2.16	1.85	1.67	1.29
21.96	99	Caryophyllene	1.21	0.87	0.79	0.73	0.52	0.87	0.85	0.82	0.67	0.98	0.93	0.85	0.69
22.37	96	Terpinen-4-ol	0.23	0.18	0.14	0.12	0.09	0.18	0.16	0.15	0.11	0.20	0.17	0.16	0.11
23.45	97	Humulene	0.52	0.35	0.31	0.23	0.17	0.35	0.33	0.31	0.22	0.45	0.38	0.32	0.23
23.82	93	Germacrene D	0.70	0.49	0.44	0.36	0.27	0.49	0.47	0.44	0.39	0.56	0.51	0.46	0.40
24.08	98	2-Cyclohexen-1-one, 3-methyl-6-(1-methylethyl)	0.07	0.07	0.06	0.05	0.04	0.07	0.06	0.06	0.05	0.01	0.07	0.06	0.05
24.95	97	4-Hexen-1-ol, 5-methyl-2-(1-methylethenyl)-acetate	0.11	0.08	0.07	0.06	0.04	0.08	0.07	0.07	0.06	0.09	0.08	0.07	0.07
			LED (μg/g)				N2+LED+CS (μg/g)								
			15 d	30 d	45 d	60 d	15 d	30 d	45 d	60 d					
6.60	97	β-Pinene	0.46	0.44	0.33	0.27	0.72	0.62	0.55	0.43					
8.41	97	β-Myrcene	1.35	1.18	1.08	0.97	2.39	2.12	1.94	1.54					
8.90	92	2-Carene	0.23	0.21	0.16	0.12	0.33	0.30	0.28	0.22					
9.68	97	D-Limonene	5.85	5.10	4.99	4.30	9.53	8.18	7.39	6.51					
9.97	94	β-Phellandrene	0.83	0.65	0.57	0.49	1.30	1.17	1.05	0.83					
10.25	97	Eucalyptol	0.83	0.76	0.07	0.05	1.38	1.21	0.11	0.09					
11.45	97	Gamma-Terpinene	0.48	0.35	0.27	0.20	0.79	0.70	0.60	0.45					
11.72	97	β-Ocimene	0.29	0.19	0.17	0.12	0.41	0.37	0.29	0.24					
17.46	98	Thujone	0.74	0.58	0.52	0.45	1.09	0.99	0.89	0.80					
18.78	94	Ethyl 2-(5-methyl-5-vinyltetrahydrofuran-2-yl)propan-2-yl carbonate	0.48	0.37	0.31	0.26	0.71	0.61	0.54	0.48					
19.87	95	Pentadecane	0.08	0.03	0.03	0.02	0.09	0.06	0.06	0.09					
21.05	87	Linalool	22.25	19.24	18.61	16.07	31.25	29.42	27.44	24.93					
21.21	91	Linalyl acetate	1.51	1.07	0.94	0.86	2.25	2.04	1.83	1.50					
21.96	99	Caryophyllene	0.68	0.6	0.58	0.53	1.15	1.01	0.93	0.78					
22.37	96	Terpinen-4-ol	0.17	0.11	0.10	0.09	0.21	0.19	0.17	0.13					
23.45	97	Humulene	0.32	0.23	0.21	0.19	0.49	0.42	0.35	0.29					
23.82	93	Germacrene D	0.40	0.33	0.31	0.29	0.65	0.57	0.48	0.42					
24.08	98	2-Cyclohexen-1-one, 3-methyl-6-(1-methylethyl)	0.08	0.05	0.04	0.04	0.07	0.07	0.06	0.54					

## Data Availability

The original contributions presented in this study are included in the article. Further inquiries can be directed to the corresponding author.
